# Identification of a 6-gene signature for the survival prediction of breast cancer patients based on integrated multi-omics data analysis

**DOI:** 10.1371/journal.pone.0241924

**Published:** 2020-11-10

**Authors:** Wenju Mo, Yuqin Ding, Shuai Zhao, Dehong Zou, Xiaowen Ding

**Affiliations:** 1 The Cancer Hospital of the University of Chinese Academy of Sciences (Zhejiang Cancer Hospital), Institute of Cancer and Basic Medicine (ICBM), Chinese Academy of Sciences, Beijing, China; 2 Department of Breast Surgery, Cancer Hospital of the University of Chinese Academy of Sciences, Beijing, China; 3 Department of Breast Surgery, Zhejiang Cancer Hospital, Hangzhou, China; Singapore General Hospital, SINGAPORE

## Abstract

**Purpose:**

To identify a gene signature for the prognosis of breast cancer using high-throughput analysis.

**Methods:**

RNASeq, single nucleotide polymorphism (SNP), copy number variation (CNV) data and clinical follow-up information were downloaded from The Cancer Genome Atlas (TCGA), and randomly divided into training set or verification set. Genes related to breast cancer prognosis and differentially expressed genes (DEGs) with CNV or SNP were screened from training set, then integrated together for feature selection of identify robust biomarkers using RandomForest. Finally, a gene-related prognostic model was established and its performance was verified in TCGA test set, Gene Expression Omnibus (GEO) validation set and breast cancer subtypes.

**Results:**

A total of 2287 prognosis-related genes, 131 genes with amplified copy numbers, 724 gens with copy number deletions, and 280 genes with significant mutations screened from Genomic Variants were closely correlated with the development of breast cancer. A total of 120 candidate genes were obtained by integrating genes from Genomic Variants and those related to prognosis, then 6 characteristic genes (CD24, PRRG1, IQSEC3, MRGPRX, RCC2, and CASP8) were top-ranked by RandomForest for feature selection, noticeably, several of these have been previously reported to be associated with the progression of breast cancer. Cox regression analysis was performed to establish a 6-gene signature, which can stratify the risk of samples from training set, test set and external validation set, moreover, the five-year survival AUC of the model in the training set and validation set was both higher than 0.65. Thus, the 6-gene signature developed in the current study could serve as an independent prognostic factor for breast cancer patients.

**Conclusion:**

This study constructed a 6-gene signature as a novel prognostic marker for predicting the survival of breast cancer patients, providing new diagnostic/prognostic biomarkers and therapeutic targets for breast cancer patients.

## Introduction

Breast cancer is one of the most frequently diagnosed female malignant tumors in the world and is also a main cause of cancer-related death to women [1]. Statistics showed that in 2018 there were 2.1 million newly diagnosed breast cancer cases, accounting for 11.6% of all the cancer cases of the year, moreover, breast cancer-caused death accounted for 6.6% of all cancer mortality [2]. Recent development in sequencing technology promotes the application of a new generation of high-throughput technologies, including second-generation sequencing technology, in resolving biological problems, especially in the treatment of human diseases [3]. Data-oriented, large-scale research model makes it possible to conduct comprehensive and multi-level research on diseases based on genomes and transcriptomes.

Studies found that breast cancer-related mutations of genes, including PIK3CA, TP53, ESR1, and ERBB2, can serve as biomarkers and facilitate a personalized treatment for breast cancer patients [4–7]. Single nucleotide variants (SNVs) and copy number variants (CNVs) are the most common mutations to genes associated with breast cancer [6, 8, 9]. Several systematic biological methods have been adopted to identify genetic biomarkers predictive of breast cancer prognosis and to construct genetic characteristics. Based on univariate and multivariate Cox proportional hazard model analysis, Xuemei Lv et al. established a 6-gene signature to predict the overall survival (OS) of patients with triple negative breast cancer [10]; Su J et al. constructed a 19-gene signature as an independent prognostic factor for breast cancer by analysis of DEGs [11]; Lai J et al. also developed 6-miRNA signature based on differentially expressed miRNAs (DEMs) [12]. Though these gene signatures have all been tested in an external independent data set, none of them are currently applied in clinical practice, suggesting that the development of clinically applicable robust gene signature still remains a great challenge and requires more effective signatures.

In recent years, breast cancer samples at different stages has been increasingly studied based on genomics, transcriptome, proteomics and metabolomics [13]. However, most studies only analyzed and described the changes at a single level rather than from a comprehensive perspective.

In this study, a systematic approach has been designed to identify genetic markers associated with breast cancer, in order to effectively screen reliable genetic markers associated with the prognosis of breast cancer. Breast cancer gene expression profiles, single nucleotide mutations, CNV data were collected from The Cancer Genome Atlas (TCGA) and Gene Expression Omnibus (GEO). Prognostic markers were screened by integrating genomics and transcriptomics data to establish a 6-gene signature, and the performance of the signature to predict patient survival was verified in internal test set and external validation set. We found that the 6 genes of the signature are involved in important biological processes and pathways of breast cancer. Gene Set Enrichment Analysis (GSEA) analysis also showed similar results, suggesting that the 6-gene signature can effectively predict the prognosis of breast cancer patients. The current findings provide a better understanding for the molecular mechanism of breast cancer prognosis.

## Materials and methods

### Data collection and processing

UCSC Cancer Browser (https://xenabrowser.net/datapages/) was used to download the TCGA RNA-Seq data (Illumina HiseqV2, version 2019-08-09), which contained 1218 cancer samples, 1268 samples with total follow-up information, and copy number variation (CNV, GISTIC2 methods, version 2019-08-09) data. SNP chips contained 1080 samples, and GDC annotation files were used to download the mutations shown in MAF files that contained 776 samples. From GEO (www.ncbi.nlm.nih.gov/geo), the GSE20685 dataset [14] incorporating 327 samples with standardized expression profiles and clinical follow-up information was downloaded on June 20, 2019. For the TCGA RNAseq data, we screened a total of 1088 tumor samples with follow-up information and randomly divided them into the training set (N = 544) or the test set (N = 544), with clinical follow-up information samples (N = 327) from GSE20685 served as an external validation set. Detailed distribution of patients’ age, survival, tumor (topography), lymph Node, tumor node metastasis (TNM), and tumor stage in the three data sets is shown in Table 1.

**Table 1:** Clinical information statistics of TCGA training datasets, TCGA validation datasets and GSE20685.

Characteristic		TCGA training datasets (n = 544)	TCGA validation datasets (n = 544)	GSE20685 (n = 327)
**Age(years)**	< = 50	176	159	209
	>50	368	385	118
**Survival Status**	Living	458	461	244
	Dead	86	83	83
**pathologic_T**	T 1	137	146	101
	T 2	320	309	188
	T 3	64	68	26
	T 4	21	20	12
**pathologic_N**	N 0	251	256	137
	N 1	187	176	87
	N 2	65	56	63
	N 3	34	42	40
**pathologic_M**	M 0	460	441	319
	M 1/ M X	84	103	8
**Tumor Stage**	Stage Ⅰ	87	96	
	Stage Ⅱ	307	308	
	Stage Ⅲ	130	115	
	Stage Ⅳ	12	9	

### Univariate Cox proportional hazard regression analysis for screening candidate genes

For the TCGA training set samples, univariate Cox regression analysis [15] was performed to investigate the relationship between OS and gene expressions of breast cancer patients. P < 0.05 was defined as statistically significant.

### Copy number variation (CNV) data analysis

GISTIC is widely used to detect both broad and focal (potential overlapping) recurring events. We used the GISTIC 2.0 software [16] to identify genes with significant amplification or deletion. The threshold for amplification and deletion was ˃ 0.1 and p < 0.05, respectively.

### Gene mutation analysis

For the MAF files of the TCGA mutation annotation data, Mutsig2 was used to screen genes with significant mutations, according to the threshold of p < 0.05.

### Functional enrichment analysis

Enrichment analysis was performed using Gene ontology (GO) and Kyoto Encyclopedia of Genes and Genomes (KEGG) and DAVID (database for annotation, visualization, and integrated discovery, see http://david.abcc.ncifcrf.gov/) [17], with P<0.05 as a threshold. Gene-set enrichment analysis (GSEA) was conducted to determine whether genes from a particular pathway or other predefined genomes were differentially expressed in different phenotypes [18]. Pathways from Reactome were analyzed by GSEA with clusterProfiler [19].

### Selection of characteristic genes

Genes with significant amplification or deletion was screened by GISTIC 2.0, and we obtained a total of 1,004 CNVs. Mutsig2 detected 280 genes with significant mutations were detected. A total of 1021 genes with significant CNV amplification, deletion or mutation were considered as genes related to genome variation. Univariate survival analysis based on genome-wide expression profiles identified 2287 genes significantly related to breast cancer prognosis. Finally, a total of 120 characteristic genes (S1 Fig) closely related to both genome variation and the prognosis of breast cancer were obtained.

### Construction of prognostic gene signature

Genes significantly associated with OS of breast cancer patients and those with amplification, deletion, and mutation were identified, and the prognosis-related genes were further ranked by randomSurvivalForest [20]. Following a previous study [21], in randomSurvivalForest, Nrep = 100, Nstep = 5, Monte Carlo iteration number was 100 and forward step number was 5, and genes had relative importance ˃ 0.25. The importance of these 120 genes was examined by randomSurvivalForest in the R Package. The number of random variables (mtry parameter) for each segmentation was set from 1 to 120, and we found that the error rate was the lowest when mtry = 6. Six genes with the highest value of relative importance and their corresponding relative importance above 0.25 were identified as characteristic genes and recruited into the construction of the final model. Multivariate Cox regression analysis was performed to construct the following risk scoring model:

$$RiskScore=\sum_{k=1}^{n}Exp_{k}*e^{HR}{}_{k}$$

Where N is the number of prognostic genes, *Exp*_*k*_ is the expression value of the prognostic genes, and *e*^*HR*^_*k*_ is the estimated regression coefficient of genes after the multivariate Cox regression analysis.

### Statistical analysis

Kaplan-Meier (KM) curves were plotted base on the median risk score from each data as a cutoff to compare the survival risk between high-risk and low-risk groups (death not due to cancer was censored). Multivariate Cox regression analysis was performed to examine whether genetic markers were independent prognostic factors. Statistical significance was defined as p <0.05. AUC analysis was carried out with pROC package, and heatmap was drawn by pheatmap in R. All the analyses were performed with default parameters in version 3.4.3 of R software, except for special instructions.

## Result

### Identification of gene sets associated with the OS of breast cancer patients

For the TCGA training set samples, Univariate regression analysis was performed to examine the relationship between patient OS and gene expression, and 2287 genes with prognostic differences were identified. Among the genes, there were 811 genes with a hazard ratio (HR) ˃ 1, and 1476 genes with an HR < 1 (S1 Table).

### Identification of genes set of genomic variation

**Fig 1:**
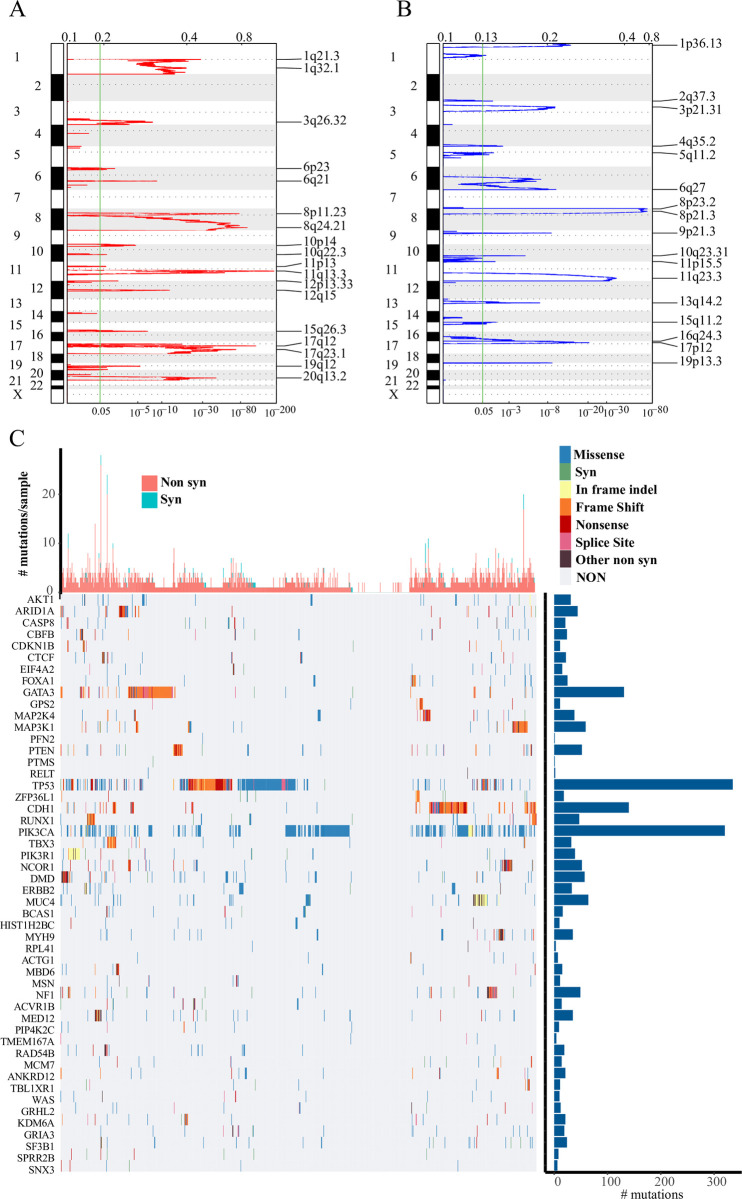
A: Significantly amplified fragments in the breast cancer genome. B: Significantly deleted fragments in the breast cancer genome. C: The distribution of the 50 most significant P genes in breast cancer patients, the top histogram shows the total number of synonymous and non-synonymous mutations in 50 genes per patient, and the right histogram shows the number of mutations in all the 50 genes. Different colors in the heat map indicate the type of mutation, and the gray color indicates no mutation.

Genes with significant amplification or deletion was screened by GISTIC 2.0, and 1,004 genes with copy number differences were detected. 131 genes were significantly amplified in the genome (Fig 1A), and had a variety of the segments closely related to the development of breast cancer. For example, CCND1 was significantly amplified in the 11q13.3 segment (q value = 2.69E-188); ERBB2 was noticeably amplified in the 17q12 segment (q value = 1.45E-118); and PIK3CA was greatly amplified in the 3q26.32 segment (q value = 1.99E-08) (S2 Table). Moreover, 724 genes showed significant deletions (Fig 1B), and some of them have been previously reported to be associated with tumorigenesis. Specifically, there was a significant deletion of CD3D in the 11q23.3 segment (q value = 8.22E-38); and CDKN2B was deleted from the 9p21.3 segment (q value = 1.95E-09); MAP3K1 was deleted from the 5q11.2 segment (q value = 0.012831) (S3 Table). Moreover, a total of 280 genes with significant mutations were identified by Mutsig2 (S4 Table). We showed that the distribution of 50 genes with the lowest P value from the TCGA breast cancer sample, noticeably those genes were accompanied with synonymous mutations, missense mutations, frame-insert or deletion, frame-shifting, nonsense mutations, shear sites, or other non-synonymous mutations (Fig 1C). The specific patterns of gene mutations varied according to different samples, in which TP53 and PIKC3A had the highest frequency of mutation, and other genes, for example, CTCF, GATA3, PTEN, CDH1, ERBB2, which are closely related to the development of breast cancer, also had high frequencies of mutations.

### Pathway and biological analysis of genomic variation-related genes

**Fig 2:**
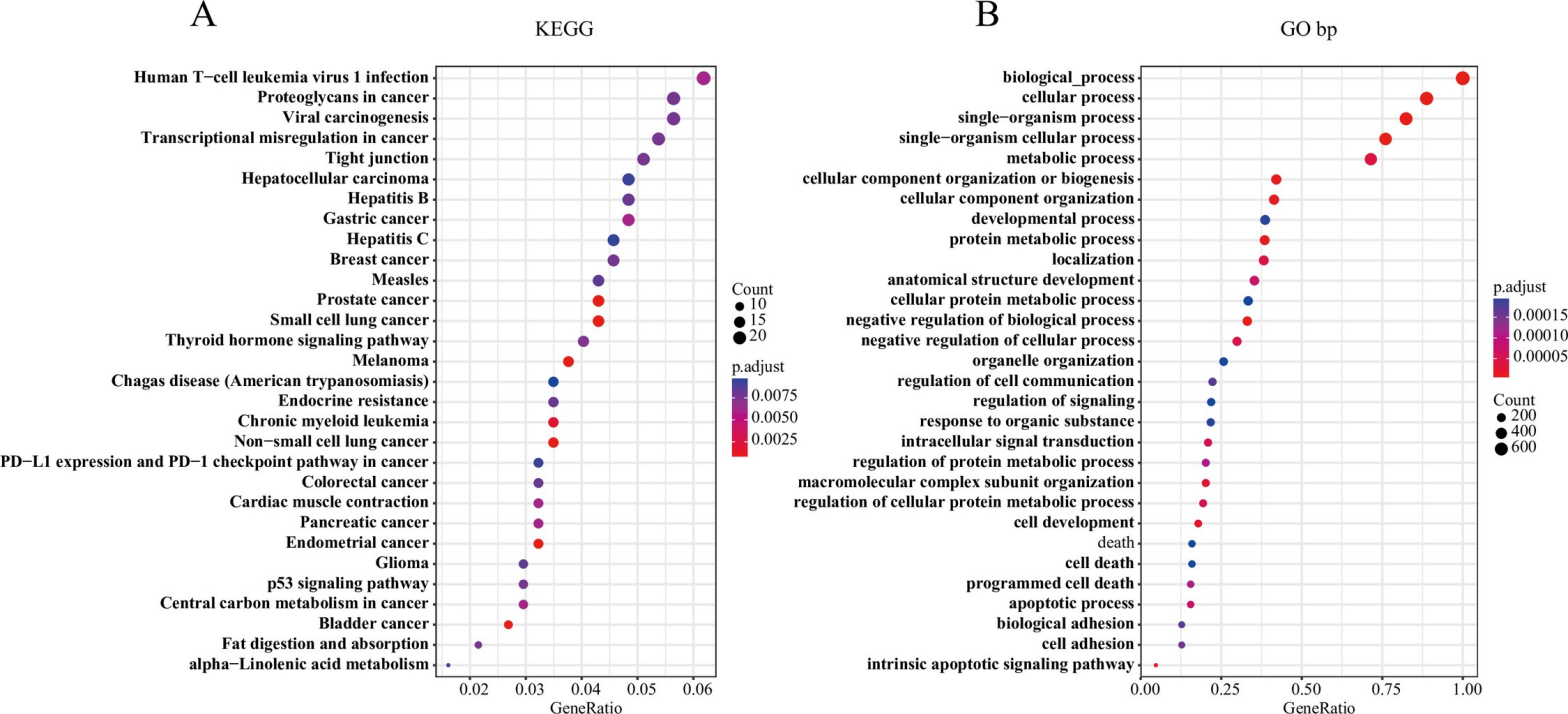
A: The 1021 genes with copy number variation and mutation are involved in the KEGG pathway. B: The 1021 genes with copy number variation and mutation are involved in the GO bp pathway.

To examine the functions of genomic mutant genes, we integrated a total of 1021 amplified and deleted genes identified by CNV with significantly mutated genes. 1021 genes were greatly enriched in KEGG pathway, which has been proven to be related to cancer genesis and development of human T-cell leukemia virus 1 infection, proteoglycans in cancer, breast cancer, p53 signaling pathway, and central carbon metabolism (Fig 2A). In the biological process category, genes were mainly enriched in cellular and metabolic processes, intracellular signal transduction, cell development, cell death and the GO Term (Fig 2B). Thus, the genes of these genomic variants were closely related to tumors.

### Establishment of a 6-gene signature for predicting BC survival

**Fig 3:**
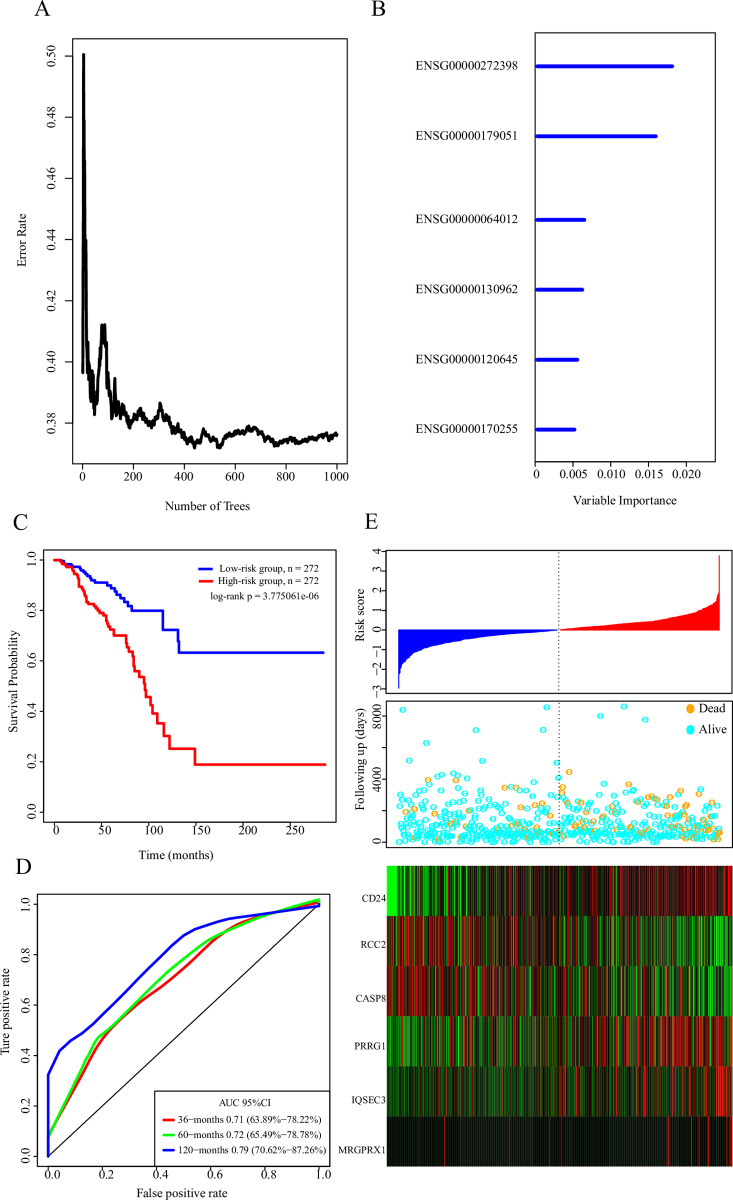
A: The relationship between the error rate and the number of classification trees. B: The importance of out-of-bag of the first 6 genes. C: KM survival curve distribution of the 6-gene signature in the TCGA training set. D: The ROC curve and AUC of the 6-gene signature classification. E: Risk score, survival time, survival status and expression of the 6 genes in TCGA training.

Here, 2287 genes related to breast cancer prognosis were screened, but only 120 genes with amplification, deletion or mutation were considered as candidate genes. The relationship between the error rate and the number of classified trees was determined by RandomForest combined with feature selection (Fig 3A). A total of 6 genes with relative importance ˃ 0.25 were recruited to establish the signature (Table 2). The importance of out-of-bag errors for the 6 genes was listed in Fig 3B. A 6-gene signature was established by performing multivariate COX regression analysis based on the following model:

$$Risk_{6}=0.4626257*CD24-0.3372725*RCC2-0.2435298*CASP8+0.1956165*PRRG1+0.159493*IQSEC3+0.1526432*MRGPRX1$$

Based on the median value (cutoff = -0.00976543) of the risk score of each sample, 272 patients were grouped into the low-risk group, whereas 272 patients were in the high-risk group. There was a significant prognostic difference between the two groups (Fig 3C). The 5-year AUC of the 6-gene signature in the training set was 0.79 (Fig 3D). The relationship between the expressions of the 6 genes and the risk score were examined, the data revealed that high-expressed CD24, PRRG1, IQSEC3 and MRGPRX1 were correlated with higher risk, suggesting that high expressions of RCC2 and CASP8 may be correlated with the low risk. Thus, RCC2 and CASP8 were considered as protective factors in breast cancer (Fig 3E).

**Table 2:** The 6 genes were significantly associated with the overall survival in the training-set patients.

Ensembl Gene ID	Symbol	HR	Z-score	P value	Importance	Relative Importance
ENSG00000272398	CD24	1.44	2.899175	3.74E-03	0.0178	1
ENSG00000179051	RCC2	0.76	-2.290401	2.20E-02	0.0157	0.878
ENSG00000064012	CASP8	0.79	-2.025316	4.28E-02	0.0062	0.3476
ENSG00000130962	PRRG1	1.29	2.695815	7.02E-03	0.0059	0.3323
ENSG00000120645	IQSEC3	1.22	2.445439	1.45E-02	0.0053	0.2957
ENSG00000170255	MRGPRX1	1.19	3.914714	9.05E-05	0.0049	0.2744

### Robustness of the 6-gene signature model

**Fig 4:**
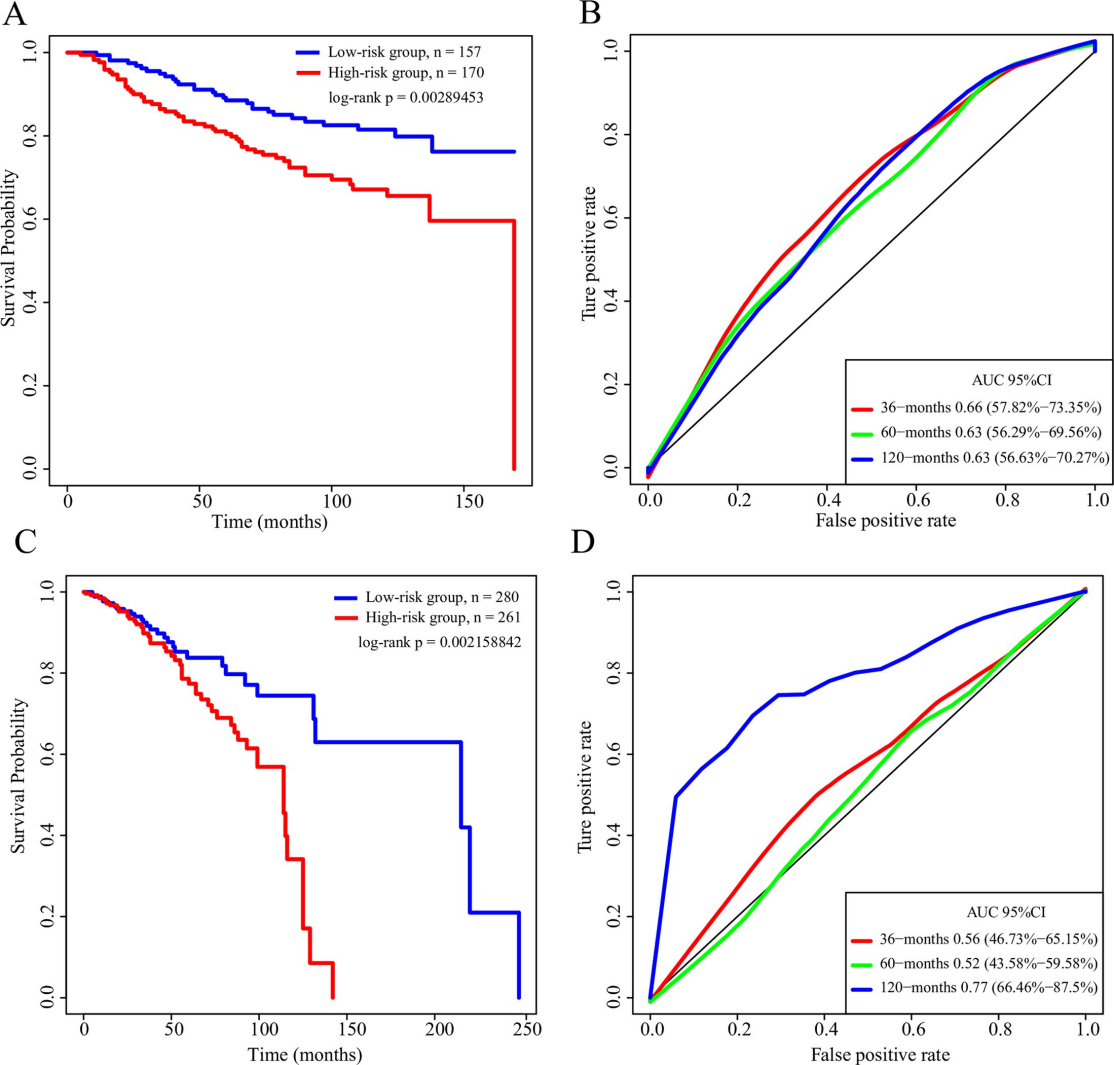
A: KM survival curve of the distribution of the 6-gene signature in the TCGA test set. B: The ROC curve and AUC of the 6-gene signature classification in the TCGA test set. C: KM survival curve distribution of the 6-gene signature in GSE20685 dataset. D: The ROC curve and AUC of the 6-gene signature classification in GSE20685 dataset.

To determine the robustness of the 6-gene signature model, we validated and calculated the risk score for each sample in the TCGA test set, and divided the samples into two groups based on the threshold of the training set. There was a significant difference in prognosis detected between the two groups (Fig 4A). The ROC curve analysis showed that the 5-year AUC was 0.63 (Fig 4B). An external validation set GSE20685 was also used for detecting robustness of the 6-gene signature model. There was a significant difference in prognosis detected between the two groups (Fig 4C), and ROC curve analysis showed that the 5-year AUC was 0.77 (Fig 4D).

### The 6-gene signature is an independent prognostic factor

In order to identify the independence of 6-gene signature model in clinical application, univariate and multivariate COX regression analysis were performed to analyze the relevant HR, 95% CI of HR, p value from the clinical information downloaded from TCGA training set, TCGA test set and GSE20685 data. We systematically analyzed the clinical information of patient records, including age, pathology T phase, N phase, M phase, and tumor Stage, from TCGA and GSE20685 as well as and the 6-gene signature grouping information (Table 3). In the TCGA training set, univariate COX regression analysis showed that high 6-gene risk score group, age, pathologic T 3, pathologic T 4, pathologic N 1, pathologic N 2, pathologic N 3, pathologic M 1/ MX, tumor stage III, tumor stage IV were significantly correlated to survival rate. The corresponding multivariate COX regression analysis demonstrated that high 6-gene risk score group (HR = 2.61, 95% CI = 1.87–3.62, p = 1.14E-08), age and tumor stage IV were independent prognostic factor. In the TCGA test set, univariate COX regression analysis showed that high 6-gene risk score group, age, pathologic T 4, pathologic N 1, pathologic N 2, pathologic N 3, tumor stage III, tumor stage IV were significantly associated with patients’ survival, but multivariate COX regression analysis indicated that high 6-gene risk score group (HR = 1.61, 95% CI = 1.11–2.31, p = 0.011) and age were independent prognostic factor. In the GSE20685, univariate COX regression analysis demonstrated that high 6-gene risk score group, pathologic T 2, pathologic T 3, pathologic T 4, pathologic N 1, pathologic N 2, pathologic N 3, pathologic M 1 and Tumor stage Ⅲ/Ⅳ were significantly correlated with the survival of breast cancer patients, and multivariate COX regression analysis found that only high 6-gene risk score group (HR = 1.83, 95%CI = 1.14–2.93, p = 0.012) and pathologic N 1 were independent prognostic factors. The above data indicated that our model 6-gene signature was an independent prognostic factor.

**Table 3:** Univariate and multivariate COX regression analysis was used to identify prognostic clinical factors and clinical independence in the TCGA training set, TCGA test set and GSE20685.

Variables	Univariate analysis			Multivariable analysis		
	HR	95%CI of HR	P value	HR	95%CI of HR	P value
**TCGA training datasets**						
6-gene risk score						
Low risk group	1(reference)			1(reference)		
High risk group	2.72	2.01–3.67	7.80E-11	2.61	1.87–3.62	1.14E-08
Age	1.03	1.01–1.04	0.003	1.03	1.01–1.05	0.001
Pathologic T 1	1(reference)			1(reference)		
Pathologic T 2	1.45	0.83–2.52	0.19	1.14	0.46–2.82	0.773
Pathologic T 3	2.04	1.00–4.14	4.90E-02	1.1	0.34–3.47	0.873
Pathologic T 4	3.61	1.54–8.41	2.97E-03	0.81	0.22–2.95	0.747
Pathologic N 0	1(reference)			1(reference)		
Pathologic N 1	2.12	1.29–3.47	0.003	1.78	0.92–3.42	0.087
Pathologic N 2	2.09	1.03–4.24	0.04	0.95	0.29–3.04	0.93
Pathologic N 3	5.29	2.25–12.39	1.25E-04	1.23	0.40–3.70	0.717
Pathologic M 0	1(reference)			1(reference)		
Pathologic M 1/ M X	2.54	1.44–4.49	1.27E-03	0.92	0.34–2.45	0.86
Tumor stage Ⅰ	1(reference)			1(reference)		
Tumor stage Ⅱ	1.53	0.76–3.06	0.2321	1.02	0.31–3.33	0.976327
Tumor stage Ⅲ	2.98	1.43–6.18	0.00341	2.67	0.54–13.16	0.227719
Tumor stage Ⅳ	15.49	6.18–38.79	4.97E-09	9.61E+00	1.55–59.44	0.01
**Validation cohort, TCGA test datasets, GSE20685**						
**TCGA test datasets**						
6-gene risk score						
Low risk group	1(reference)			1(reference)		
High risk group	1.54	1.13–2.08	0.006	1.605	1.11–2.31	0.011
Age	1.03	1.02–1.05	4.15E-05	1.038	1.01–1.05	1.91E-04
Pathologic T 1	1(reference)			1(reference)		
Pathologic T 2	1.12	0.65–1.93	0.676	1.23	0.51–2.95	0.638
Pathologic T 3	1.12	0.54–2.29	0.764	0.983	0.32–2.97	0.976
Pathologic T 4	4.43	2.06–9.53	1.36E-04	3.308	0.96–11.34	0.057
Pathologic N 0	1(reference)			1(reference)		
Pathologic N 1	1.83	1.03–3.24	0.038	1.312	0.62–2.73	4.68E-01
Pathologic N 2	3.8	1.90–7.55	0	2.973	0.82–10.67	9.49E-02
Pathologic N 3	3.37	1.47–7.68	0.004	2.317	0.62–8.54	2.07E-01
Pathologic M 0	1(reference)			1(reference)		
Pathologic M 1/ M X	1.43	0.84–2.42	1.87E-01	0.442	0.13–1.46	0.182
Tumor stage Ⅰ	1(reference)			1(reference)		
Tumor stage Ⅱ	1.48	0.70–3.09	0.299	1.29	0.37–4.49	0.686
Tumor stage Ⅲ	2.74	1.25–5.96	0.011	1.2	0.21–6.65	0.835
Tumor stage Ⅳ	8.42	3.09–22.88	3.00E-05	7.718	0.80–73.70	0.076
**GSE20685**						
6-gene risk score						
Low risk group	1(reference)			1(reference)		
High risk group	1.89	1.36–2.62	1.22E-04	1.83	1.14–2.93	0.012
Age	0.99	0.97–1.01	4.83E-01	0.99	0.97–1.01	0.285
Pathologic T 1	1(reference)			1(reference)		
Pathologic T 2	1.14	0.66–1.94	6.42E-01	1.00	0.48–2.10	0.997
Pathologic T 3	4.81	2.44–9.43	5.29E-06	1.65	0.63–4.31	0.311
Pathologic T 4	4.43	1.94–10.07	3.89E-04	0.87	0.20–3.77	0.847
Pathologic N 0	1(reference)			1(reference)		
Pathologic N 1	2.41	1.23–4.66	9.62E-03	3.50	1.28–9.56	0.015
Pathologic N 2	5.11	2.73–9.48	2.77E-07	0.64	0.13–3.06	0.579
Pathologic N 3	5.11	2.54–10.22	4.38E-06	0.61	0.13–2.93	0.544
Pathologic M 0	1(reference)			1(reference)		
Pathologic M 1	5.21	2.39–11.33	3.22E-05	2.63	0.72–9.66	0.145
Tumor stage Ⅰ	1(reference)			1(reference)		
Tumor stage Ⅱ	0.97	0.46–2.06	0.938	0.39	0.11–1.46	0.163
Tumor stage Ⅲ/Ⅳ	3.97	2.01–7.81	6.83e-05	4.59	0.70–29.86	0.111

### GSEA analysis on differences of enriched pathway between high-risk group and low-risk group

Significantly enriched pathways in the high-risk group and the low-risk group in the TCGA training set were analyzed by performing GSEA. The selected gene set was c2.cp.kegg.v6.0.symbols, which was involved in the KEGG pathways.

**Fig 5:**
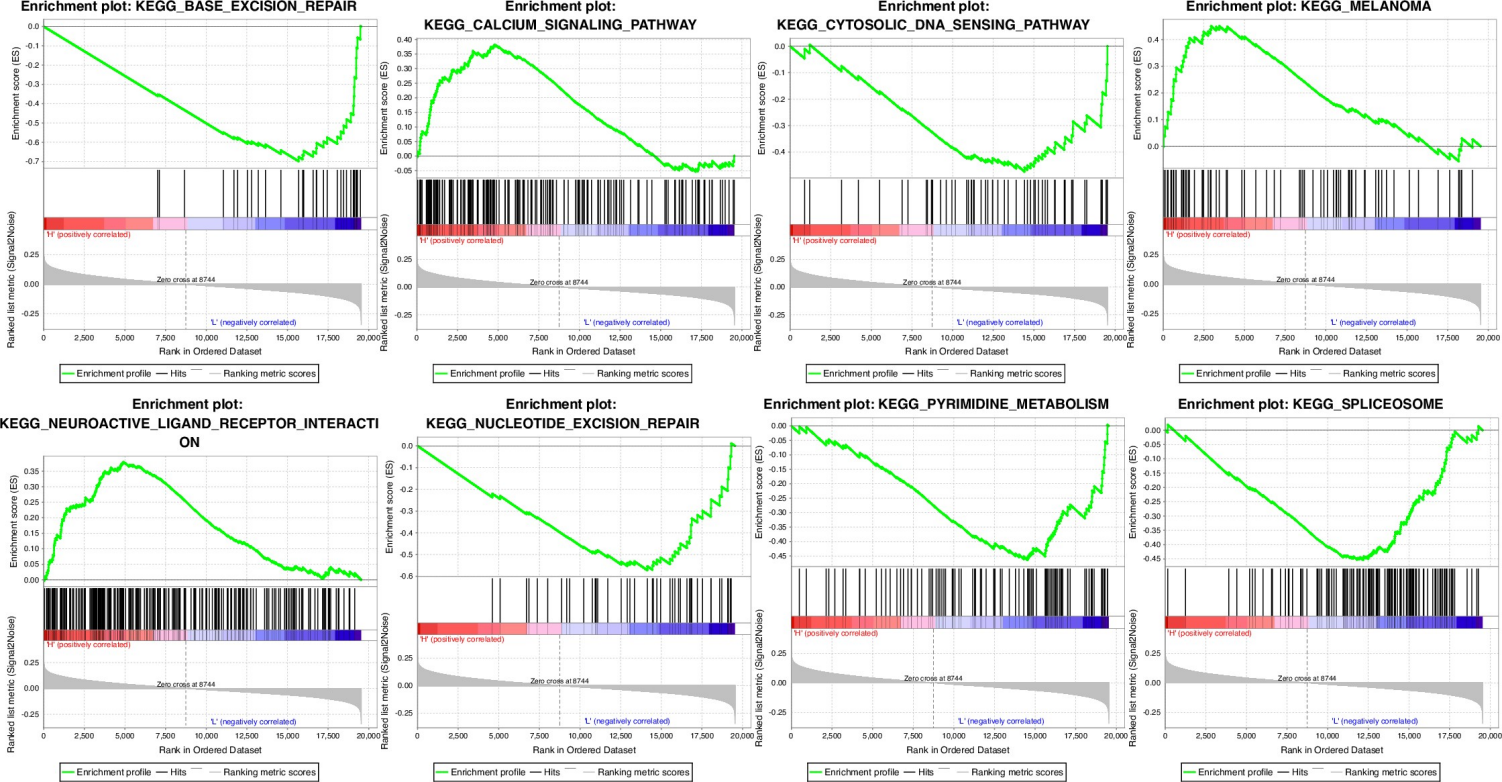
KEGG_BASE_EXCISION_REPAIR, KEGG_MELANOMA, KEGG_PYRIMIDINE_METABOLISM, KEGG_NUCLEOTIDE_EXCISION_REPAIR, KEGG_SPLICEOSOME, KEGG_CALCIUM_SIGNALING_PATHWAY, KEGG_NEUROACTIVE_LIGAND_RECEPTOR_INTERACTION and KEGG_CYTOSOLIC_DNA_SENSING_PATHWAY GSEA enrichment results for eight pathways.

The GSEA inputfile contained the expression spectrum data normalized by the TCGA training set, and the sample label of the 6-gene signature was used to assign the sample into high-risk group or low-risk group. The significantly enriched pathway was obtained according to the threshold of p<0.05 (S5 Table). Noticeably, the pathways, namely, KEGG_BASE_EXCISION_REPAIR, KEGG_MELANOMA, KEGG_PYRIMIDINE_METABOLISM, KEGG_NUCLEOTIDE_EXCISION_REPAIR, KEGG_SPLICEOSOME, KEGG_CALCIUM_SIGNALING_PATHWAY, KEGG_NEUROACTIVE_LIGAND_RECEPTOR_INTERACTION and KEGG_CYTOSOLIC_DNA_SENSING_PATHWAY were significantly enriched in the high-risk and low-risk groups and were also closely associated with the development and metastasis of breast cancer (Fig 5).

### Robustness of the 6-gene signature model in breast cancer subtypes

**Fig 6:**
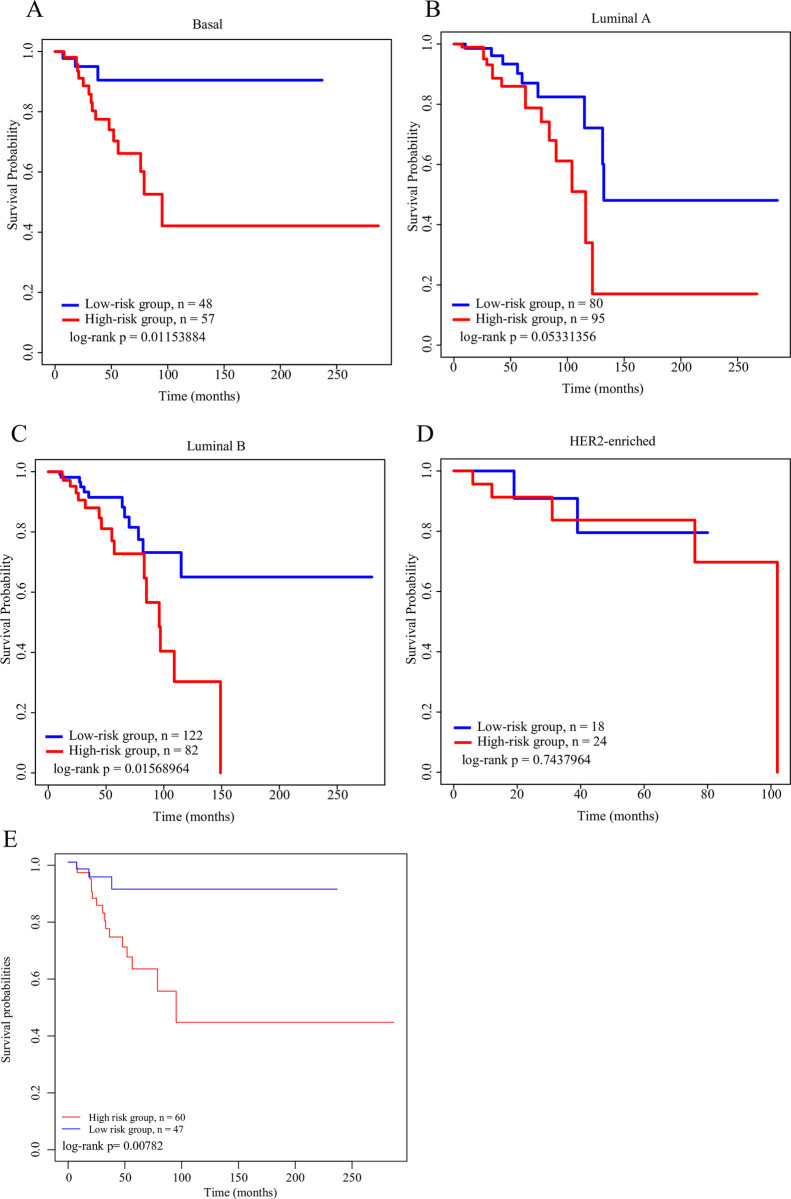
A: KM survival curves of the distribution of the 6-gene signature in breast cancer subtypes Basal. B: KM survival curves of the distribution of the 6-gene signature in breast cancer subtypes Luminal A. C: KM survival curves of the distribution of the 6-gene signature in breast cancer subtypes Luminal B. D: KM survival curves of the distribution of the 6-gene signature in breast cancer subtypes Her2-enriched. E: KM survival curves of the distribution of the 6-gene signature in breast cancer subtypes triple negative breast cancer.

In the TCGA training set, breast cancer samples including 105 samples of Basal subtypes, 42 samples of Her2-enriched subtypes, and 175 samples of Luminal A subtypes and 204 samples of Luminal B subtypes, were divided into different molecular subtypes according to the pam50 method (S6 Table) into the high- and low-risk groups. We found that the corresponding log-rank values of Basal, Luminal A, Luminal B and triple negative breast cancer models were 0.01153884, 0.05331356, 0.01568964 and 0.0078, respectively, indicating the robustness of the models (Fig 6A–6C and 6E). In the Her2-enriched subtype, the high- and low-risk groups could not be effectively divided (Fig 6D), indicating that our model was applicable to most molecular subtypes, but its applicability to Her-enriched was relatively poor. In order to examine the relationship between drug therapy and the model, the clinical information of drug therapy was downloaded using R software TCGAbiolinks and analyzed in the training set sample. The results showed that non-treated patients with high risk had the worst prognosis, while those with low risk and treatment developed the most favorable prognosis (S2A Fig). Further analysis showed that our 6-gene model was able to precisely distinguish untreated patients from the high- and low-risk groups, but the same results were not observed in the treated patients (S2B and S2C Fig).

## Discussion

Breast cancer is one of the most common malignant tumors among women worldwide [22]. The treatments for breast cancers, including resection, chemotherapy and radiotherapy, are constantly advancing, but their limited sensitivity and specificity lead to largely poor prognosis of patients with the cancer [23, 24]. Screening prognostic molecular markers fully indicative of the biological characteristics of breast cancer plays an important role in individualized prevention and treatment for breast cancer patients. In this study, we analyzed the expression profiles of breast cancer samples from TCGA and GEO using multiple omics data, and constructed a robust 6-gene signature independent of clinical factors for predicting the OS of breast cancer patients.

Studies on gene signatures in cancers are increasingly emerging. Based on univariate and multivariate Cox proportional hazard model analysis, Xuemei Lv et al. also established a 6-gene signature to predict the OS of triple negative breast cancer [10]; Foulds GA et al. analyzed a subset of 23 randomly selected breast cancer patients from the NanoString nCounterTM platform to construct a 3-gene signature, which can predict the relapse of triple negative breast cancer [25]; an artificial neural network-derived 3-gene signature has also been developed to improve the accuracy of stratifying acute myeloid leukemia patient stratification [26]; moreover, a 8-gene signature has been constructed based on macrophage-breast cancer cell interactions [27]. In the bioinformatics analysis of breast cancer, the AUC of our 6-gene signature was close to 0.7 in the training set, test set and verification set, and the 6 genes all showed abnormal genome mutations, which facilitate clinical diagnosis of breast cancer. These data suggested that our 6-gene signature had a high AUC with fewer genes involved, showing a great potential in clinical transformation.

In 6-gene signature, high expressions of CD24, PRRG1, IQSEC3, and MRGPRX1 were risk factors correlated with high risk, while high expressions of RCC2, CASP8 were protective factors correlated with low risk. It has been reported that CD24 expression is heterogeneous in tumors [28, 29]. The expressions of the miR-17-92 cluster members and host gene in HCC tissues are negatively related to the expressions of several target genes, including that of PRRG1 [30]. Study showed that overexpression of RCC2 promotes cell movement and induces tumor metastasis of lung adenocarcinoma by inducing epithelial-mesenchymal transition [31]. Lymph node metastasis is associated with specific hotspot somatic mutations in TP53 and CASP8 [32]. IQSEC3 and MRGPRX1 are tumor-related genes and were first discovered in this study as a new prognostic marker for breast cancer. Moreover, our GSEA analysis revealed that pathways to which the 6-gene signature was enriched were also closely related to the biological processes of breast cancer development. Nucleotide excision repair and Base_excision_repair are two major pathways for the repair of DNA crosslink caused by cisplatin in cancer including breast cancer [33]. In vitro and in vivo metabolic profiling using triple-negative breast cancer cells suggests that increased abundance of pyrimidine nucleotides occurs in response to chemotherapy exposure [34]. Studies have suggested that the spliceosome component may be a therapeutic entry point for aggressive MYC-driven cancers [35]. Calcium_signaling_pathway, neuroactive_ligand_receptor_interaction and cytosolic_DNA_sensing_pathway are involved in breast cancer development [36–38] The melanoma pathway is an important cancer pathway that involves the cell cycle, transcriptional activation, and PI3K signaling pathway (https://www.genome.jp/kegg-bin/show_pathway?hsa05218), and it's well known that cell cycle and transcriptional activation are closely linked to tumorigenesis. These results suggested that the model can provide a potential target for clinical diagnosis of breast cancer.

Although we identified potential candidate genes for predicting the prognosis of breast cancer through bioinformatics with a large sample size, some limitations of this study should also be noted. First, our sample lacked clinical follow-up information, therefore factors such as the presence of other health conditions were not considered during the identification of prognostic biomarkers. Also the results obtained only through bioinformatics analysis were less convincing, which requires further experimental validation. Moreover, further genetic and experimental studies based on a larger sample size and experimental validation are also needed.

In conclusion, the current study developed a 6-gene signature prognostic stratification system with high AUC in both training set and validation set and was independent of clinical features. Compared with the clinical features, gene classifier can improve the accuracy in predicting the survival risk of patients. Therefore, we recommend using this classifier as a molecular diagnostic tool to evaluate the prognostic risk of breast cancer patients.

## Supporting information

S1 Fig (TIF)

S2 Fig (TIF)

S1 Table (XLSX)

S2 Table (TXT)

S3 Table (TXT)

S4 Table (TXT)

S5 Table GSEA analyzed significantly enriched KEGG pathways in high-risk and low-risk groups. (DOCX)

S6 Table (TXT)
