# Supplementary figures and images for: Identification of a 6-gene signature for the survival prediction of breast cancer patients based on integrated multi-omics data analysis
Source: PLoS One. 2020 Nov 10;15(11):e0241924. doi: 10.1371/journal.pone.0241924 (PMC7654770; doi:10.1371/journal.pone.0241924)

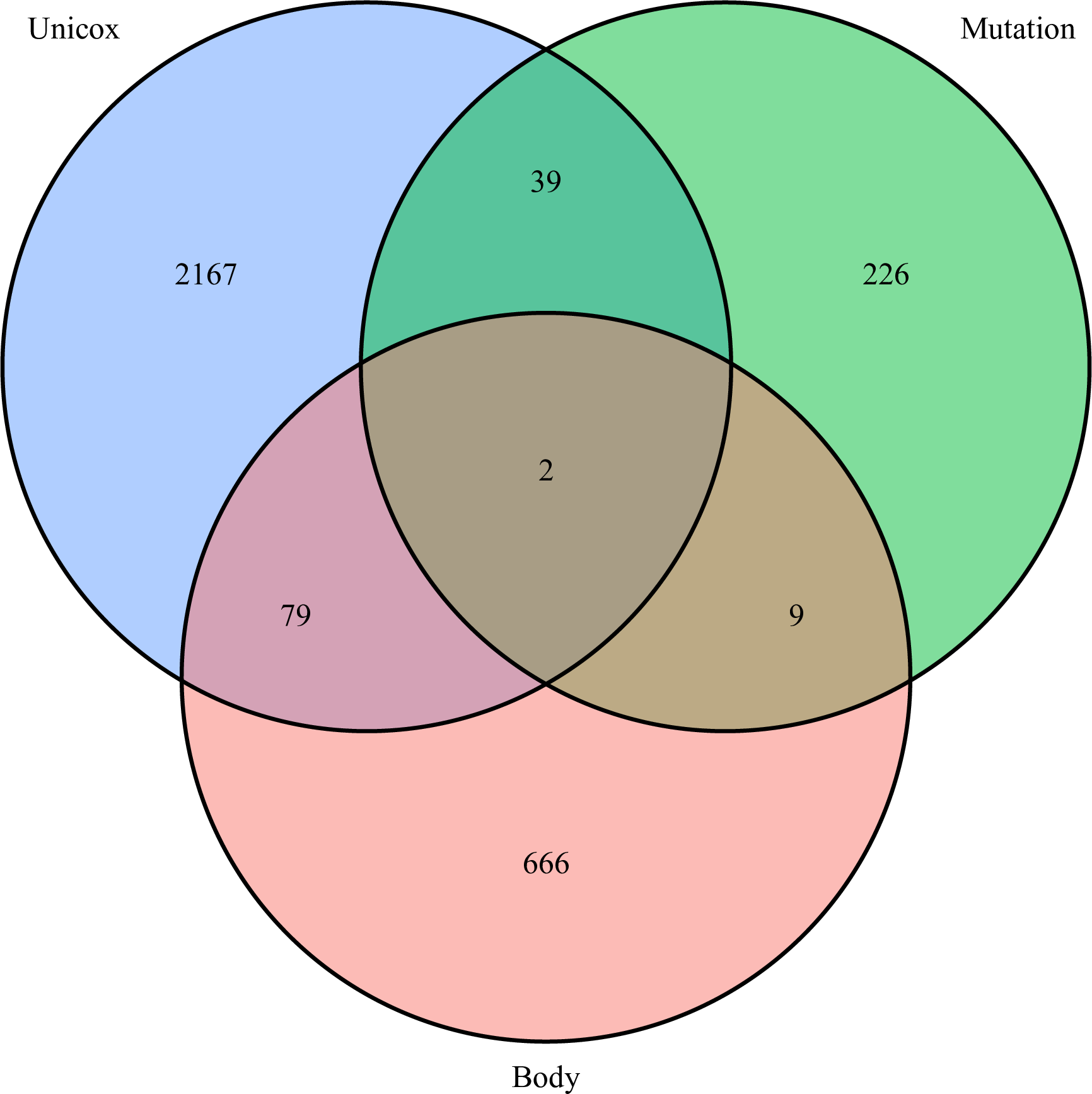

Supplement: S1 Fig — (TIF) [file pone.0241924.s001.tif]

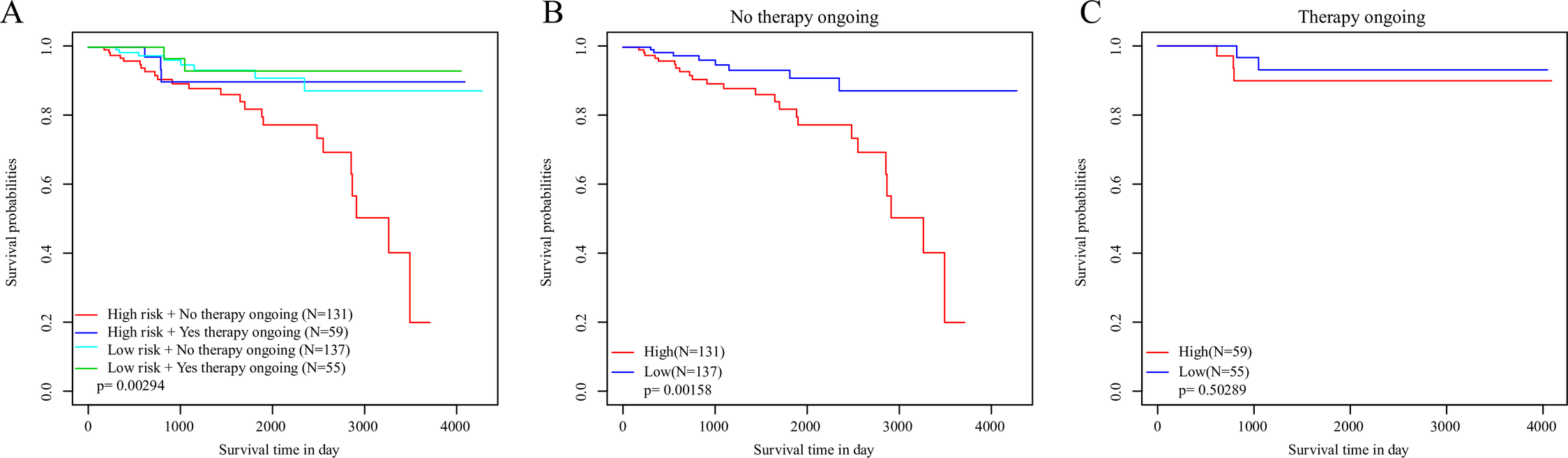

Supplement: S2 Fig — (TIF) [file pone.0241924.s002.tif]
