# Supplementary material for: Identification of a 6-gene signature for the survival prediction of breast cancer patients based on integrated multi-omics data analysis
Source: PLoS One. 2020 Nov 10;15(11):e0241924. doi: 10.1371/journal.pone.0241924 (PMC7654770; doi:10.1371/journal.pone.0241924)
Supplement: S5 Table — (DOCX) [file pone.0241924.s007.docx]

**S5 Table.** GSEA analyzed significantly enriched KEGG pathways in high-risk and low-risk groups.

| **Name** | **Size** | **ES** | **NES** | **NOM p-val** | **FDR q-val** | **FWER p-val** |
| --- | --- | --- | --- | --- | --- | --- |
| KEGG_BASE_EXCISION_REPAIR | 33 | -0.703 | -1.943 | 0.002 | 0.141 | 0.104 |
| KEGG_MELANOMA | 71 | 0.427 | 1.681 | 0.006 | 1.000 | 0.586 |
| KEGG_PYRIMIDINE_METABOLISM | 95 | -0.472 | -1.727 | 0.010 | 0.320 | 0.516 |
| KEGG_NUCLEOTIDE_EXCISION_REPAIR | 44 | -0.577 | -1.809 | 0.011 | 0.314 | 0.317 |
| KEGG_SPLICEOSOME | 123 | -0.564 | -1.730 | 0.021 | 0.418 | 0.511 |
| KEGG_CALCIUM_SIGNALING_PATHWAY | 175 | 0.362 | 1.512 | 0.023 | 1.000 | 0.884 |
| KEGG_NEUROACTIVE_LIGAND_RECEPTOR_INTERACTION | 271 | 0.356 | 1.495 | 0.027 | 0.641 | 0.900 |
| KEGG_CYTOSOLIC_DNA_SENSING_PATHWAY | 54 | -0.471 | -1.562 | 0.045 | 0.447 | 0.836 |
